# Beyond VDR: Vitamin D Metabolism Genes in Tuberculosis Susceptibility, Immune Response and Implications for Diabetes Mellitus

**DOI:** 10.2147/IDR.S616991

**Published:** 2026-08-10

**Authors:** Marrita Rabadi, Zaeem Hussain, Sundus Farooq Siddiqui, Hiba Farooq Siddiqui, Michail Nomikos, Mohammed Seed Ahmed

**Affiliations:** 1 College of Medicine, QU Health, Qatar University, Doha, Qatar

**Keywords:** vitamin D metabolism, cytochrome P450, tuberculosis, diabetes mellitus, immune response

## Abstract

**Background:**

Vitamin D is an important immunomodulator in host defense against *Mycobacterium tuberculosis*, yet most tuberculosis-related research has focused on the vitamin D receptor (VDR). The contribution of vitamin D metabolism genes, particularly cytochrome P450 (CYP) enzymes, remains less clearly defined.

**Methods:**

We conducted a narrative review examining the role of major vitamin D–metabolizing enzymes, including CYP2R1, CYP27A1, CYP27B1, and CYP24A1, in tuberculosis susceptibility, immune regulation, and treatment response, with additional focus on diabetes mellitus as a major comorbidity. Evidence from genetic association studies, observational studies, mechanistic experiments, and clinical data was synthesized.

**Results:**

Vitamin D metabolism enzymes influence intracellular vitamin D activation and inactivation, affecting antimicrobial peptide production, autophagy, cytokine signaling, and macrophage-mediated clearance of *M. tuberculosis*. Genetic and epigenetic variation in these pathways may contribute to differences in tuberculosis risk and prognosis. Diabetes mellitus may further disrupt these mechanisms through chronic hyperglycemia, oxidative stress, inflammation, impaired macrophage and T-cell function, and alterations in vitamin D metabolism, potentially increasing tuberculosis susceptibility and worsening outcomes. However, direct evidence for several enzymes remains limited. Inconsistent findings from vitamin D supplementation trials suggest that circulating 25(OH)D may not accurately reflect functional vitamin D activity at infection sites. Current evidence is largely mechanistic, observational, and genetic, with few interventional studies.

**Conclusion:**

Vitamin D metabolism genes represent an underexplored but potentially important component of tuberculosis pathophysiology, particularly in patients with diabetes. Future work should prioritize genetically stratified host-directed approaches to identify patients most likely to benefit from vitamin D–based interventions.

## Introduction

TB remains one of the leading causes of death from infectious disease worldwide. According to the WHO Global Tuberculosis Report 2025, TB claimed more than 1.2 million lives and affected an estimated 10.7 million people globally in the previous year.^1^ High TB-burden regions with widespread vitamin D deficiency include countries in Sub-Saharan Africa (South Africa, Nigeria, Kenya), South Asia (India, Pakistan, Bangladesh), Southeast Asia (Indonesia, the Philippines, Myanmar),^2^ and some parts of the Middle East (Saudi Arabia, Iran).^3^ Although notable advances have been achieved in diagnosis and treatment, persistent funding gaps and inequities in access to care continue to pose significant threats to sustaining and accelerating progress in global TB control.^4^

Diabetes mellitus (DM), a rapidly growing global health burden, is a recognised risk factor for tuberculosis. It increases the probability of active TB by roughly two to three times and is associated with poorer clinical outcomes, such as delayed sputum conversion, higher relapse, and mortality.^5–7^ According to new research, vitamin D insufficiency is very common in people with diabetes and may be a factor in their compromised immune systems.^8–10^ In addition, patients with type 2 diabetes who have low serum vitamin D levels have decreased monocyte function, which reduces their ability to prevent MTB from growing intracellularly.^11^ Additionally, diabetes may have an impact on vitamin D metabolism itself, especially in the circulating levels of vitamin D and the enzyme CYP24A1,^12^ although the influence of other vitamin D metabolism genes in TB–DM comorbidity remains poorly understood.

Vitamin D-mediated immunity has been extensively studied in tuberculosis (TB), primarily through the lens of the vitamin D receptor (VDR). However, emerging evidence indicates that upstream regulation of vitamin D activity governed by vitamin D metabolism enzymes, particularly cytochrome P450 (CYP) enzymes play a critical yet underexplored role in host defense against *Mycobacterium tuberculosis*.

**Figure 1:**
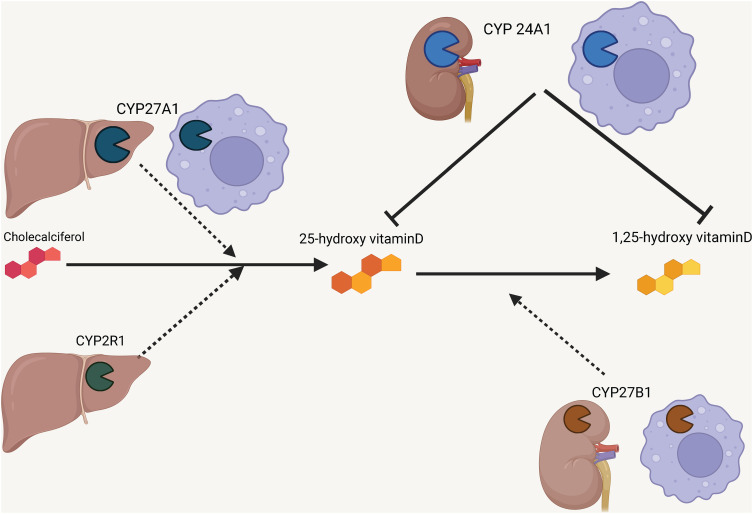
Overview of vitamin D metabolic pathways and key cytochrome P450 enzymes involved in immune modulation. Hepatic CYP2R1 and CYP27A1 convert cholecalciferol to 25-hydroxyvitamin D [25(OH)D]. In the kidney and immune cells, CYP27B1 further converts 25(OH)D to the biologically active form, 1,25-dihydroxyvitamin D [1,25(OH)_2_D]. CYP24A1 regulates vitamin D availability both locally and systemically by mediating the degradation of active vitamin D metabolites. Solid arrows (→) indicate reaction direction; dashed arrows (-->) indicate enzymatic catalysis; blunt-ended (⊣) arrows indicate enzymatic breakdown or degradation. Created in BioRender. Alrabadi, (M) (2026) https://BioRender.com/bx7x0ij.^14^

Vitamin D is a fat-soluble prohormone obtained from dietary sources and synthesized in the skin upon exposure to ultraviolet B radiation. In the liver, it undergoes hydroxylation to form 25-hydroxyvitamin D [25(OH)D], the main circulating form and standard marker of vitamin D status. This intermediate is further hydroxylated in the kidneys to produce the biologically active form, 1,25-dihydroxyvitamin D [1,25(OH)_2_D], which binds to the vitamin D receptor (VDR) to regulate calcium and phosphate homeostasis, bone metabolism, as well as forming a heterodimer with retinoid-X receptor (RXR) in immune cells upon binding, and activating antimicrobial peptides such as cathelicidin and β-defensin 2, which enhances pathogen clearance.^13^ Unlike renal metabolism, which is regulated by systemic calcium, phosphate, and parathyroid hormone, extra-renal activation of vitamin D is primarily controlled by immune signals, including cytokines and pathogen-associated molecular patterns, allowing vitamin D to directly modulate immune responses at sites of infection or inflammation^14^ (Figure 1).

**Figure 2:**
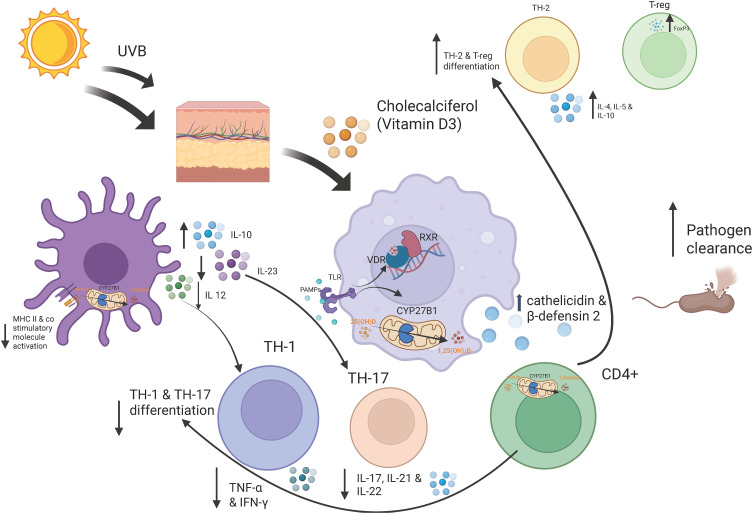
Vitamin D–mediated immune modulation and antibacterial defense. Ultraviolet B (UVB) exposure induces cutaneous synthesis of vitamin D_3_, which is subsequently converted to 25-hydroxyvitamin D [25(OH)D] and locally activated in immune cells by CYP27B1 to the biologically active form, 1,25-dihydroxyvitamin D [1,25(OH)_2_D]. Toll-like receptor (TLR) signaling enhances pathogen clearance by upregulating VDR–RXR activation and inducing antimicrobial peptides, including cathelicidin and β-defensin-2. Vitamin D suppresses Th1 and Th17 differentiation and reduces pro-inflammatory cytokines (eg, IL-12 and IL-23), while promoting anti-inflammatory IL-10 production. Concurrently, Th2 and regulatory T cell (Treg) responses are enhanced, contributing to immune homeostasis and controlled inflammation. Upward arrows (↑) indicate upregulation and increased expression, whereas downward arrows (↓) indicate downregulation and decreased expression. Created in BioRender. Alrabadi, (M) (2026) https://BioRender.com/ol3p4oz.^15^

In dendritic cells (DCs), 1,25(OH)_2_D downregulates MHC class II and co-stimulatory molecules (CD40, CD80, CD86) and suppresses cytokine production, including IL-12 and IL-23, thereby limiting Th1 and Th17 differentiation while promoting the anti-inflammatory cytokine IL-10. Monocytes and macrophages recognize pathogens via toll-like receptors, which upregulate the vitamin D receptor (VDR) and CYP27B1. 1,25(OH)_2_D suppresses CD4⁺ T cell proliferation, reduces Th1- and Th17-type cytokines (IL-2, IFNγ, TNF-α, IL-17, IL-21, IL-22), and promotes Th2 and regulatory T cell differentiation, increasing anti-inflammatory cytokines (IL-4, IL-5, IL-10) and FoxP3 expression. Together, these actions enable vitamin D to fine-tune both innate and adaptive immunity.^15^ (Figure 2).

As an important immunomodulator, vitamin D deficiency is common in regions with high tuberculosis (TB) prevalence, with significant clinical implications. Deficiency of vitamin D has been associated with increased susceptibility to active TB, severity of disease, and delayed response to therapy.^16^ Addressing vitamin D deficiency may represent a cost-effective adjunct to TB prevention and treatment strategies in these populations.^16^

Despite extensive research on VDR polymorphisms and circulating vitamin D levels in tuberculosis, the contribution of vitamin D metabolism genes, including CYP27B1, CYP24A1, CYP27A1 and CYP2R1, remains comparatively overlooked. Genetic and functional variation in these enzymes may critically influence intracellular vitamin D activation, macrophage-mediated immunity, and susceptibility to *Mycobacterium tuberculosis*, yet existing findings are fragmented and sometimes inconsistent.

Importantly, no review to our knowledge has comprehensively synthesized the role of non-VDR vitamin D metabolism genes in tuberculosis while simultaneously considering the modifying role of diabetes mellitus. Because diabetes-related immunological dysfunction and altered vitamin D metabolism may interact to worsen TB outcomes, this constitutes a major knowledge gap. This review presents a thorough and current synthesis of the role of vitamin D metabolism enzymes in TB susceptibility, immunological modulation, and clinical consequences, with a focus on its implications in diabetic populations.

## Susceptible Vitamin D Metabolic Enzymes

### CYP2R1

CYP2R1 is the microsomal enzyme responsible for converting cholecalciferol (vitamin D_3_) into 25-hydroxyvitamin D.^17^ The immunological effects of CYP2R1 are through eventually increasing 1,25(OH)_2_D levels in the serum. 1,25(OH)_2_D enhances antimicrobial responses to *M. tuberculosis* by induction of IL-1β in vitro.^18^ Its effects go beyond macrophages also since it drives epithelial production of antimicrobial peptides such as DEFB4/HBD2.^19^

**Figure 3:**
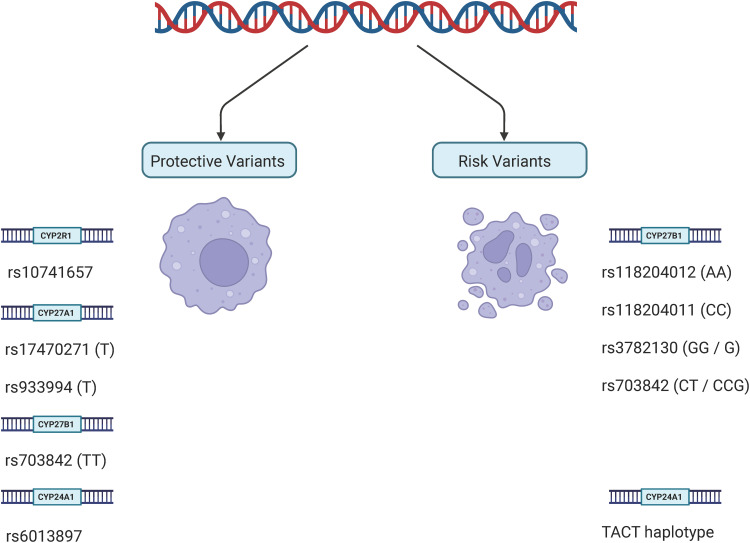
Genetic variation in vitamin D metabolism pathways and its potential impact on tuberculosis susceptibility. Genetic variants in key vitamin D metabolism genes may confer either increased risk or protection against tuberculosis. Risk-associated variants could be linked to impaired vitamin D activation, reduced antimicrobial responses, and in some cases increased susceptibility to *Mycobacterium tuberculosis*. In contrast, protective variants are associated with enhanced macrophage function, improved vitamin D bioavailability, and more effective pathogen clearance. The diagram illustrates how host genetic variation along the vitamin D pathway modulates immune responses to *Mycobacterium tuberculosis*. Created in BioRender. Alrabadi, (M) (2026) https://BioRender.com/mck84rp.^22^

Based on this biological rationale, studies were conducted to see the effect of CYP2R1 gene mutations on TB susceptibility. Although there have been conflicting studies most of them have shown no significant difference in TB outcomes.^20–22^ Some genetic studies find an impact of CYP2R1 mutations on TB outcome, but no mechanism independent of VDR-mediated immunity has been established for CYP2R1 mutations in influencing TB prognosis.^23^ For example, A study conducted in India found that rs10741657 gene polymorphisms were associated with increased levels of serum Vitamin D and protection against TB (Figure 3), however this study did not determine whether the association was independent of VDR polymorphisms.^24^ On the other hand, another study conducted in pregnant women with latent TB found no change in vitamin D levels associated with the rs10741657 gene polymorphism and therefore no impact on TB prognosis.^25^

Even though its mechanism of improving prognosis in TB is to increase serum vitamin D levels, which is sound, the current evidence remains inconclusive due to the small number of studies conducted. Therefore, it is difficult to directly link polymorphisms in the CYP2R1 gene to TB prognosis independent of other steps in the pathway, such as VDR, which means that its effects cannot be linked to any immune cell intrinsic effects since the enzyme is mainly intrahepatic. This is also because there have not been many studies conducted that investigate the effects of this enzyme independent of VDR. To our knowledge, the precise impact of diabetes or dysglycemia on CYP2R1 activity in relation to tuberculosis is still unknown.

### CYP27A1

CYP27A1 is a mitochondrial enzyme that is mainly expressed in macrophages and the liver. It helps to catalyze the 25-hydroxylation of vitamin D, which is then converted to the active form 1,25-dihydroxy-vitamin D. There is evidence to suggest that CYP27A1 plays a role in regulating the immune system by preventing cholesterol accumulation itself in macrophages or by its oxysterol products, such as 27-hydroxycholesterol and 3β-hydroxy-5-cholestenoic acid, acting on the LXR receptors.^26^

In TB specifically, a study of the IL-36/LXR axis in macrophages infected with *Mycobacterium tuberculosis* found that knockdown of CYP27A1 impaired LXR induction and led to increased bacterial growth. In other words: CYP27A1 contributed to the macrophage’s ability to contain Mycobacterium TB.^27^ Furthermore, another mechanism of the enzyme’s beneficial effects is through oxysterols generated by CYP27A1 contributing to reverse cholesterol transport in the lung, which may influence immunity during Mtb infection.^28^ Therefore, in TB, the effect of CYP27A1 appears to extend beyond its classical role in vitamin D metabolism, and therefore VDR, since the role of CYP27A1 in increasing serum vitamin D levels is not as significant, it’s primary mechanism of improving immunity is through its role in the cholesterol pathway. It’s role in cholesterol metabolism contributes to host-directed immunity through LXR-mediated regulation of lipid homeostasis and inflammatory signaling in macrophages, which together helps in the intracellular control of *Mycobacterium tuberculosis* infection.

Genetic studies have also found a link in CYP27A1 gene variations and prognosis of tuberculosis. A mixed case-control and prospective cohort study from China examined genes involved in the vitamin D metabolic pathway, as vitamin D intervention studies yielded inconsistent results. The study found an interaction between CYP27A1 and the association of vitamin D concentration with TB risk. This means that individuals with certain methylation profiles may derive less immunological benefit from vitamin D, thereby increasing the risk of TB.^23^ Another study found that CYP27A1 polymorphisms were significantly associated with severe clinical features of pulmonary TB but were not associated with disease susceptibility. For the enzyme’s gene, they also found that the rs17470271 T allele frequency was significantly lower in patients with leukopenia, and the rs933994 T allele frequency was significantly decreased in patients with drug resistance, yet no significant results were found for variants rs17470271 and rs933994 in developing pulmonary TB.^29^ A case-control study found that CYP27B1 SNP rs10877012 showed deviation from Hardy-Weinberg Equilibrium with statistical significance. This may reflect disease association or population structure.^22^ Overall, there is some evidence to suggest that certain methylation profiles of the enzyme’s variants may be protective against TB, yet it is unknown how diabetes or dysglycemia specifically affects CYP27A1-mediated pathways in tuberculosis.

### CYP27B1

CYP27B1 encodes the enzyme 25-hydroxyvitamin D-1α-hydroxylase, which converts circulatory 25-hydroxyvitamin D (25(OH)D) into 1,25-dihydroxyvitamin D (1,25(OH)_2_D), the active form. It is mainly located in the kidney and immune cells, such as macrophages. Macrophages express CYP27B1 to locally produce 1,25(OH)_2_D as part of the host response to infection (Figure 2). *Mycobacterium tuberculosis* components that activate the Toll-like receptor (TLR), leading to upregulation of CYP27B1 in monocytes, which raises 1,25(OH)_2_D levels and downstream vitamin D receptor (VDR) signaling. The expression of cathelicidin (LL-37) and the activation of autophagy, two important processes by which macrophages eliminate *M. tuberculosis*, depend on CYP27B1 induction for complete antimycobacterial activity, as demonstrated by a study showing that TLR2/1 stimulation with a mycobacterial lipoprotein requires these steps. Therefore, CYP27B1 deficiency or downregulation can potentially hinder immune cell production of 1,25(OH)_2_D, resulting in decreased autophagy and cathelicidin production, and ultimately decreased macrophage capacity to control TB infection.^30^,^31^ To the best of our knowledge, no research specifically investigated how diabetes or dysglycemia affect CYP27B1 activity in patients with tuberculosis.

One study found no association between a CYP27B1 variant and TB susceptibility. In particular, a CYP27B1 SNP, rs4646536, was not associated with increased susceptibility to pulmonary TB in a Chinese population. Nonetheless, a family-based study demonstrated that both CT and TT genotypes of this SNP could increase susceptibility to vitamin D deficiency as compared to the CC genotype.^32^

However, recent studies have identified certain CYP27B1 polymorphisms that may increase TB susceptibility. In a case-control study conducted in Chennai, South India, CYP27B1 polymorphisms were assessed; two main polymorphisms were noteworthy. Regarding rs118204011, the CC genotype was most frequent, and for rs118204012, the AA genotype was most common. Of note, the rs118204012 “G” allele appeared protective for TB (Figure 3). Dominant model analysis confirmed a protective effect of the AG/GG genotypes compared to AA. Additionally, gender-specific analysis indicated that males with rs118204011 “CC” or rs118204012 “AA” genotypes were at significantly higher risk of TB than females, suggesting a sex-related genetic predisposition and vitamin D insufficiency was more common in these^33^ (Table 1).

**Table 1:** Genetic Variations in Genes Linked to Vitamin D Metabolism and Their Reported Correlations with Tuberculosis Clinical Outcomes and Susceptibility

Reference	Variant (rs/Promoter/ Haplotype)	Risk Genotype/Allele (Worse TB)	Protective Genotype/Allele (Better TB)	Association with TB	Sample Size
CYP2R1					
[33]	rs10741657	—	Allele associated with higher vitamin D levels	Possible protective effect (inconsistent evidence)	126
CYP27A1					
[29]	rs17470271	—	T allele	Reduced leukopenia (better prognosis)	979
[29]	rs933994	—	T allele	Reduced drug resistance (better prognosis)	979
[22]	rs10877012	–	–	May be associated with disease or population structure	84
CYP27B1					
[33]	rs118204012	AA	G allele (AG/GG)	Increased TB susceptibility in some alleles	126
[33]	rs118204011	CC	—	Increased TB susceptibility	126
[29]	rs4646536	CT / TT (linked to vitamin D deficiency)	CC	No direct TB association	979
[33]	rs3782130 (−1077 C/G)	GG, G allele CCG	—	Increased TB risk	126
[33]	rs10877012 (−1260 C/A)	CCG	—	No significant association	
[33]	rs703842 (−1918 C/T)	CT CCG	TT	CT increases risk, TT protective	
CYP24A1					
[22]	rs6013897	—	Variant allele	Decreased TB susceptibility	84
[29]	Multi-SNP haplotype “TACT”	TACT haplotype	—	Increased TB susceptibility	979

A further investigation in the South Indian population aimed to assess whether CYP27B1 gene promoter variants- rs3782130 (−1077 C/G), rs10877012 (−1260 C/A), and the rs703842−1918 C/T variant located immediately 5’ to the promoter- are associated with pulmonary tuberculosis. The study utilized various models to capture distinct effects of homozygous and heterozygous alleles on disease susceptibility. The models used were co-dominant (analyzing each genotype separately), dominant (assessing the effect of a risk allele), and over-dominant (evaluating heterozygote effects). The study found that the −1077G allele and −1077 “GG” genotype were associated with increased TB risk (co-dominant model: OR 1.52, p = 0.025; OR 2.10, p = 0.015) but showed a protective effect in the dominant model (OR 0.40, p = 0.0035). The −1918 “CT” genotype conferred TB risk (over-dominant: OR 1.93, p = 0.047), whereas −1918 “TT” showed a protective effect (recessive: OR 0.43, p = 0.046) (Figure 3). The −1077 “GG” variant reduced CYP27B1 expression via Nuclear Factor 1 (NF-1) binding, while −1260C altered 1α-hydroxylase activity. However, no association was observed for −1260 in their cohort. Additionally, haplotype analysis revealed that “CCG” (−1918C, −1260C, −1077G) increased TB risk (OR 10.26, p = 0.017), and strong linkage disequilibrium was observed between −1077 and −1260 (D’ = 0.93), with “GC” haplotype associated with higher TB susceptibility (OR 11.39, p = 5.43×10^−5^). Sex-stratified analysis was also performed, indicating higher TB risk in males with the −1077, −1260, and −1918 risk genotypes, consistent with previous findings for CYP2R1 and CYP27B1 variants.^24^ Furthermore, promoter variants showed trends toward lower vitamin D in risk genotypes (−1077 “GG,” −1260 “CA,” −1918 “CT”), with higher prevalence of vitamin D deficiency among patients. Mechanistically, these variants may downregulate CYP27B1 expression via altered transcription factor binding or promoter hypermethylation, contributing to immune dysfunction.^33^ These results imply that vitamin D metabolism and tuberculosis susceptibility are influenced by variations in the CYP27B1 gene and promoter. Hence, this emphasises the importance of considering sex-specific and genetic characteristics when analysing host immunological responses to tuberculosis and could help guide focused approaches to disease management and prevention.

### CYP24A1

The 25-hydroxyvitamin D-24-hydroxylase, the primary enzyme responsible for breaking down active vitamin D, is encoded by CYP24A1. By initiating the breakdown of 1,25(OH)_2_D into inactive metabolites, it blocks vitamin D signalling. The fact that CYP24A1 is a vitamin D-responsive gene is significant because it creates a negative feedback loop to stop excessive vitamin D activity in cells when 1,25(OH)_2_D binds to VDR.^34^ Hence, an increased expression or activity of CYP24A1 can cause the immunological response to be reduced by the rapid clearance of 1,25(OH)_2_D. On the other hand, the active vitamin D can last longer and have longer-lasting immunological effects if CYP24A1 activity is inhibited.^35^,^36^

Despite its complex actions, vitamin D is generally considered to exert anti-inflammatory effects overall.^37^,^38^ It is noteworthy to mention that the primary degradation pathway mediated by CYP24A1 is thought to be impaired in macrophages, as human macrophages appear to partially express a nonfunctional splice variant of this enzyme (CYP24A1-SV). Additionally, the metabolism of 25-hydroxy-vitamin D in macrophages is heavily dependent on their polarization state. In essence, human macrophages metabolize vitamin D differently depending on whether they are in an inflammatory (M1 macrophages) or anti-inflammatory (M2 macrophages) state. While M2 macrophages continually express mRNA for the CYP27B1 enzyme in order to synthesize vitamin D, M1 macrophages synthesize only a small amount of active vitamin D and upregulate the expression of CYP24A1-SV. Consequently, M2 macrophages secrete high levels of 1,25-dihydroxy-vitamin D while M1 macrophages demonstrate almost no secretion of active vitamin D.^37^

When 25(OH)D was examined in human keratinocytes (OKF6/TERT2 cells), CYP24A1 inhibition increased cathelicidin (LL-37) levels and enhanced bacterial death. In a comprehensive genotyping study of a Chinese population that examined several genes involved in the vitamin D pathway, individual SNPs in CYP24A1 were not significantly associated with TB susceptibility. However, haplotype analysis showed that TB patients had a higher frequency of a particular multi-SNP combination in CYP24A1 (haplotype “TACT”) than healthy controls. This risk haplotype was associated with higher odds of tuberculosis, indicating that specific combinations of CYP24A1 genetic variants may collectively enhance tuberculosis risk, even if individual variants have little to no impact.^29^

One case control study conducted in Kazakhstan reported that the rs6013897 variant of the CYP24A1 enzyme may confer protective effects against the development of TB (OR = 0.436, CI 0.191–0.996, p = 0.045) (Figure 3). In this study, the investigators analyzed control participants who cohabited with individuals diagnosed with tuberculosis. Despite prolonged exposure within the same household environment, these controls remained uninfected. This provides a rigorous framework for assessing host susceptibility, suggesting that inherent genetic factors may play a critical role in mediating resistance to *Mycobacterium tuberculosis*.^22^ Conversely, the comprehensive genotyping study conducted in China revealed that this same allele was not associated with protection against nor susceptibility to TB.^29^ It is proposed that this allele functions in the context of other genetic variations in the vitamin D pathway, potentially affecting serum vitamin D levels or the subsequent biochemical signalling necessary for the activation of immune-related genes, which could partially explain the discrepancy observed between these two studies.^22^

In addition to genetic polymorphisms, epigenetic modifications to the CYP24A1 gene may also influence the risk of developing TB. A case-control study conducted on a Chinese Han population revealed hypomethylation of the CYP24A1 gene may be associated with an increased risk of developing diabetes in TB as a comorbidity, suggesting a potential role for CYP24A1 in the pathological process. However, no correlation was observed between CYP24A1 methylation and glycemic markers, such as glucose or HbA1c, suggesting that altered CYP24A1 activity may primarily influence susceptibility to TB through immune-related mechanisms rather than through effects on hyperglycemia. In this sample, serum 25-hydroxyvitamin D (25(OH)D) levels were significantly higher in healthy individuals than in patients with tuberculosis and diabetes.^39^ These results are especially important in the setting of diabetes, where host immunological responses to Mycobacterium TB may be compromised by both epigenetic changes and impaired vitamin D metabolism. Another study based in China reciprocated the aforementioned findings, further demonstrating that hypomethylation of CYP24A1 gene and lower serum levels of vitamin D was associated with increased risk of TB development and, subsequently, poorer prognosis.^23^ Although these results point to a possible link between TB susceptibility and CYP24A1 hypomethylation, there is a number of methodological issues to be considered. Because both studies were observational case-control studies carried out in Chinese populations, their generalisability was limited.^23^,^39^ Conclusions on causality or temporal associations could not be drawn, as methylation was assessed at a single point in time. Furthermore, it is yet unknown if the identified methylation alterations are a result of or a cause of diabetes-tuberculosis comorbidity, tuberculosis, or associated inflammatory processes. Additionally, there was minimal functional validation that correlated CYP24A1 methylation with vitamin D metabolism or gene expression. To further understand the role of CYP24A1 epigenetic regulation in diabetes and tuberculosis, longitudinal studies with functional analyses are required.

## Diabetes, Tuberculosis, and Vitamin D Metabolism: A Converging Axis

Diabetes may potentially have a physiological impact on the processes involved in vitamin D metabolism, however, the exact effects of diabetes on the majority of vitamin D metabolism enzymes are still poorly understood, with CYP24A1 being the primary enzyme for which preliminary diabetes-related data are currently available, yet direct mechanistic evidence remains limited.

It has been demonstrated that a pathway linking diabetes and tuberculosis is through chronic hyperglycemia modifying key pathways that substantially overlap with vitamin D-mediated immunological systems, such as altering T-cell and macrophage function, decreasing the synthesis of antimicrobial peptides, and dysregulating cytokine responses.^40^ In parallel, studies in patients with type 2 diabetes consistently report a high prevalence of vitamin D deficiency. For example, a study reported lower circulating 25(OH)D levels in 74.4% of patients with type 2 diabetes mellitus, while deficiency was found in 36.6% of these patients.^41^ Studies have attributed this to two main causes: first, vitamin D deficiency may contribute to systemic inflammation; second, vitamin D participates in insulin signaling through vitamin D receptor–mediated actions in pancreatic β-cells, liver, muscle, and adipose tissue, and activates PPAR-δ, where it may improve insulin secretion, enhance insulin sensitivity, and promote glucose homeostasis, thus its deficiency may impair such processes.^41–44^ Additionally, a cross-sectional study demonstrated that vitamin D deficiency was significantly associated with metabolic syndrome and several of its individual components, including central obesity, hyperglycemia, hypertension, and dyslipidemia.^45^

Experimental data from streptozotocin-induced diabetic rats showed that increased renal Cyp24A1 expression is accompanied by reduced circulating 1,25-dihydroxyvitamin D levels^40^. Although these results imply that diabetes may affect the catabolism of vitamin D, their relevance to tuberculosis in humans is not fully known. A mechanistic framework linking diabetes and tuberculosis is supported by evidence demonstrating that chronic hyperglycemia impairs both innate and adaptive immune responses. The accumulation of advanced glycation end products, oxidative stress, and metabolic inflammation caused by hyperglycemia all hinder T-cell activation, antigen presentation, macrophage phagocytosis, while vitamin D deficiency may further exacerbate hyperglycemia-induced immune dysfunction, and thereby intracellular destruction of Mycobacterium TB.^40^,^46^

Furthermore, Vitamin D is associated with a number of cellular and inflammatory pathways. Vitamin D deficiency has been associated with increased oxidative stress and activation of inflammatory pathways, including the NLRP3 inflammasome. In a randomized clinical trial involving 68 patients with type 2 diabetes, vitamin D3 supplementation significantly reduced NLRP3 gene expression, supporting a role for vitamin D in modulating chronic inflammation and oxidative stress.^47^ Vitamin D may also exert antioxidant effects through maintenance of intracellular reactive oxygen species homeostasis and enhancement of glutathione availability, thereby limiting oxidative stress associated with chronic hyperglycemia.^41^,^43^ Vitamin D also regulates cytokine pathways by suppressing pro-inflammatory mediators such as TNF-α, IL-1β, and IL-6 while promoting a more balanced immune response.^38^,^48^ VDR signalling activation increases autophagy, modifies innate immune responses, and encourages the synthesis of antimicrobial peptides including β-defensin 2 and cathelicidin, all of which are critical for host defence against mycobacterial infections.^48^ Besides that, by inhibiting pro-inflammatory cytokines, lowering Th1 and Th17 responses, and encouraging regulatory T-cell differentiation and IL-10 production, vitamin D affects adaptive immunity.^48^ Vitamin D further regulates cytokine pathways by suppressing pro-inflammatory mediators such as TNF-α, IL-1β, and IL-6 while promoting a more balanced immune response.^38^,^48^ Given that hyperglycemia, oxidative stress, and chronic inflammation could contribute to impaired macrophage function, dysregulated cytokine signaling, and reduced host defense against *Mycobacterium tuberculosis*, vitamin D deficiency and potential alterations in vitamin D metabolism could further exacerbate these abnormalities, providing a potential biological link between diabetes, immune dysfunction, and increased tuberculosis susceptibility.^38^,^40^,^47–50^

Vitamin D supplementation has been studied as an adjuvant treatment for tuberculosis in patients with diabetes. Since randomised controlled trials have shown variable effects on sputum culture conversion and clinical outcomes, current clinical guidelines do not advocate routine high-dose vitamin D treatment for all TB patients.^51–54^ According to a Cochrane systematic review, vitamin D does not consistently improve TB treatment outcomes, although supplementation is generally safe.^55^ Importantly, individuals with diabetes may require tailored host-directed therapeutic approaches, due to possible decreased vitamin D metabolism and immunological dysfunction, especially in cases of poor glycemic control.^56^

Therefore, although there is growing evidence that vitamin D metabolism plays a part in the comorbidity of diabetes and tuberculosis, the role of vitamin D metabolism genes in diabetic populations is still largely unexplored, creating a significant research gap. Overall, tuberculosis outcomes are worse when immune dysfunction, altered vitamin D metabolism, and metabolic changes in diabetes are compounded. This underscores the necessity for combination treatment approaches that target both immunological and metabolic pathways.

## Therapeutic Strategies

**Figure 4:**
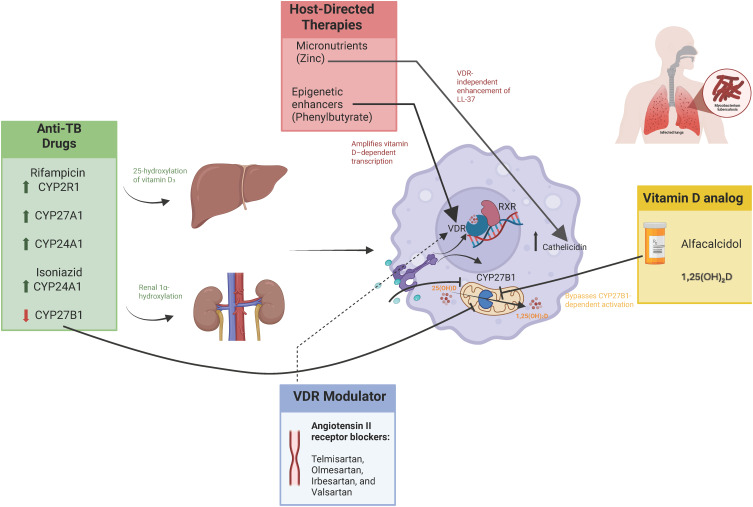
Vitamin D–macrophage axis modification for therapeutic purposes in tuberculosis. Anti-TB medications reduce intracellular 1,25(OH)_2_D via changing the metabolism of vitamin D in the liver and kidneys, boosting CYP2R1/CYP27A1 while inhibiting CYP27B1 and activating CYP24A1. Alfacalcidol, an active vitamin D analogue, restores VDR signalling by overriding CYP27B1. Mycobacterial clearance is also improved by host-directed treatments such as zinc and phenylbutyrate, which increase cathelicidin (LL-37) and autophagy. Theoretically, ARBs are suggested VDR modulators. To strengthen antimycobacterial immunity, these treatments work together to target several nodes in the vitamin D–macrophage pathway. Solid arrows (→) represent established vitamin D–dependent or biologically supported mechanisms. Dashed arrows (-->) represent theoretical or indirect interactions (eg, in silico VDR modulation). Blunt ended arrows (⊣) indicate inhibition or enzymatic bypass. Upward arrows (↑) indicate induction or increased activity of the indicated enzyme or pathway, whereas downward arrows (↓) indicate inhibition, downregulation, or reduced activity. Created in BioRender. Alrabadi, (M) (2026) https://BioRender.com/rudggrs.^59–63^

As a supplement to conventional anti-TB regimens, host-directed therapies (HDTs) that improve antimycobacterial immunity without specifically targeting *Mycobacterium tuberculosis* have received increased interest, as it can lower the risk of drug resistance.^57^ A key immunoregulatory pathway in tuberculosis is the vitamin D–macrophage axis, which combines autophagy, local vitamin D metabolism, pathogen recognition, and antimicrobial peptide induction. In addition to supplemental vitamin D, a number of pharmacologic and immunologic interventions, which include VDR activation, CYP27B1 induction, downstream effector amplification (such as cathelicidin),^58^ and interactions with anti-TB medications, could impact this axis at several levels (Figure 4).

### Vitamin D Analog: Alfacalcidol as a Potential Method of Overcoming CYP27B1

Alfacalcidol (1α-hydroxyvitamin D_3_) is a synthetic vitamin D analogue that does not require renal CYP27B1 activity because it quickly undergoes hepatic 25-hydroxylation to yield active 1,25(OH)_2_D.^59^ Pharmacologic management of active vitamin D levels is clinically possible, as evidenced by its widespread use in treating mineral and bone problems in patients with chronic renal disease.^60^,^64^,^65^

Alfacalcidol may theoretically compensate for decreased CYP27B1 activity caused by genetic variations or drug-induced inhibition during TB treatment. To our knowledge, there are no experimental or clinical investigations that explicitly assessed it in TB. Therefore, alfacalcidol’s potential advantages are still hypothetical.

### VDR Modulator: Evidence at the Theoretical Level for Angiotensin Receptor Blockers

Based on in silico docking and biochemical investigations demonstrating binding to the VDR ligand-binding domain, angiotensin II receptor blockers (ARBs) have been proposed as potential VDR modulators. For example, Telmisartan showed the best expected antagonism of the vitamin D receptor (VDR). Olmesartan, Irbesartan, and Valsartan also showed expected VDR antagonism. In conjunction with VDR, Telmisartan was projected to be a potent modulator of peroxisome proliferator-activated receptor-γ (PPARγ).^61^ However, these findings are derived primarily from computational modeling and receptor-binding studies and have not been validated in tuberculosis-specific experimental systems. To date, no human nor animal research has assessed ARBs as modulators of vitamin D signaling in tuberculosis, and there is no evidence of either immunologic benefit or antimycobacterial efficacy in this setting. Therefore, any proposed role of ARBs in tuberculosis remains hypothetical and requires further experimental and clinical investigation.

### Host-Directed Therapies

#### Epigenetic Enhancers (Phenylbutyrate)

In macrophages and epithelial cells, phenylbutyrate (PBA), a short-chain fatty acid and histone deacetylase inhibitor, increases the transcription of antimicrobial genes.^63^ Through a process that depends on vitamin D signalling and the induction of cathelicidin (CAMP/LL-37), PBA causes autophagy and limits bacterial replication in human macrophages infected with Mtb. Most importantly, PBA amplifies rather than bypasses vitamin D-dependent antimicrobial mechanisms, as proven by the requirement for an intact VDR pathway for PBA-mediated upregulation of CAMP.

According to in vitro research, PBA and 1,25(OH)_2_D work together to significantly increase LL-37 expression and autophagic elimination, thereby improving intracellular killing of Mtb.^66^ A randomised controlled trial in pulmonary tuberculosis has demonstrated translational relevance, with adjunctive PBA and vitamin D3 supplementation accelerating sputum culture conversion in comparison to placebo, indicating improved early bacterial clearance.^67^ According to these results, PBA is among the most clinically advanced HDTs that target the vitamin D–macrophage axis.

#### Micronutrients and Downstream Effectors (Zinc)

Zinc is a crucial trace element that has a role in both innate and adaptive immunity, including the control of antimicrobial peptides.^62^,^68^ Zinc increases LL-37 expression in immunological and epithelial cells through MAPK-dependent signalling pathways, especially by activating p38 and ERK,^69^ which are two pathways converge on transcription factors and downstream MAPK-activated protein kinases, allowing for post-translational regulation of immune responses and rapid adjustment of gene expression.^70^ Even though this impact appears largely independent of direct VDR activation, increased LL-37 availability can indirectly enhance vitamin D-mediated antimicrobial responses.

The findings of zinc supplementation clinical trials for tuberculosis have been inconsistent, with some studies showing a minor effect on sputum conversion or clinical outcomes, while others reveal significant benefits.^71^,^72^ For instance, compared to a placebo, an Indonesian study found that taking zinc (15 mg) plus vitamin A (5000 IU) daily for six months resulted in earlier sputum smear conversion, although the result was not statistically significant.^73^

These differences are likely due to variations in baseline nutritional status, concurrent micronutrient deficiencies, and study design. Zinc should therefore be regarded as a physiologically feasible, yet to be clinically proven, supplement that targets the vitamin D pathway’s downstream effectors.

#### Drug–Gene Interactions: Implications for Host-Directed Therapy in TB

Apart from VDR, genetic variations in vitamin D metabolism genes may affect how individuals react to such treatments. Although there is limited direct clinical evidence associating the CYP27B1 genotype to a variable response to vitamin D therapy in tuberculosis, similarities can be seen in other illnesses. A CYP27B1 promoter polymorphism (−1260 base, rs10877012) was notably linked to lower 1,25D and a worse response to interferon-α therapy in chronic hepatitis C.^74^

This implies that host immunological treatments may be hindered by insufficient vitamin D activation. Therefore, patients with tuberculosis who have a CYP27B1 variant that affects 1α-hydroxylase activity may respond differently to host-directed therapies targeting the vitamin D axis. For instance, individuals may benefit more from active vitamin D analogues (such as calcitriol or 1α-hydroxyvitamin D) that do not require 1α-hydroxylation than from ordinary vitamin D_3_ pills. In vitro studies demonstrating that giving active 1,25D to infected macrophages inhibits *M. tuberculosis* growth and triggers antimicrobial pathways highlight the significance of CYP27B1.^75^. Adjunct treatments like phenylbutyrate may also have a lesser effect if a patient’s macrophages are genetically incapable of performing this conversion, unless they are used in conjunction with active vitamin D.^63^

A case report shows that rifampicin can restore vitamin D homeostasis in patients with biallelic CYP24A1 loss-of-function mutations by initiating an alternative metabolic route via CYP3A4 to compensate for deficient CYP24A1-mediated vitamin D catabolism. These patients have reduced inactivation of 25-hydroxyvitamin D and 1,25-dihydroxyvitamin D due to defective CYP24A1 activity, which causes hypercalcemia, hypercalciuria, and suppressed parathyroid hormone levels. When rifampicin was administered, calcium metabolism returned to normal, parathyroid hormone levels rose, and urine calcium excretion decreased; these effects reversed when the medication was stopped. Increased synthesis of 4β,25-dihydroxyvitamin D_3_, a metabolite unique to CYP3A4, was validated by biochemical profiling, indicating that rifampicin-mediated vitamin D inactivation is mechanistically based on CYP3A4 stimulation.^76^ These results demonstrate that pharmacologic modulation of vitamin D metabolic enzymes can significantly alter vitamin D signalling, offering a robust mechanistic framework for understanding the potential effects of anti-tuberculosis medications like rifampicin on host-directed therapies that rely on intact vitamin D metabolism.

### Anti-TB Drug Effects on Vitamin D Metabolism

The conventional anti-tuberculosis medications themselves can significantly alter vitamin D metabolism, potentially affecting host immunity. Combination therapy with rifampicin (RIF) and isoniazid (INH) lowers circulating vitamin D levels, according to years of clinical research. In one study, RIF (600 mg) plus INH (300 mg) daily for two weeks resulted in a 34% decrease in 25(OH)D and a 23% decrease in 1,25(OH)_2_D levels in healthy volunteers (both significant). After a month of treatment, TB patients on RIF/INH displayed a similar pattern and a compensatory increase in parathyroid hormone.^77^ These medications were thought to accelerate the breakdown of vitamin D or otherwise disrupt its homeostasis by inducing liver enzymes. In fact, more recent research in mice confirms that vitamin D metabolism is significantly altered by RIF, a strong inducer of cytochrome P450 enzymes. Serum 25-hydroxyvitamin D_3_ and inactive 24,25-dihydroxyvitamin D_3_ rose in mice given RIF (± INH) for three weeks, but the active 1,25(OH)_2_D_3_ did not alter. The hepatic vitamin D-25-hydroxylases (CYP2R1 and CYP27A1) were upregulated by rifampicin, which explained this paradoxical increase in 25(OH)D_3_. In parallel, the combination of RIF and INH reduced renal CYP27B1 expression and increased the catabolic 24-hydroxylase (CYP24A1).^78^

In other words, rifampicin (especially when combined with isoniazid) appears to divert vitamin D metabolism towards inactive forms: it increases the production of 25(OH)D and 24,25(OH)_2_D, which may create the appearance of adequate 25(OH)D levels, but it hinders the conversion to 1,25(OH)_2_D within the kidneys and macrophages. Infected tissues would ultimately have fewer levels of vitamin D readily available, which would weaken the immunological response mediated by vitamin D. This occurrence has been seen without significant changes in serum calcium, but it raises the possibility that, while maintaining normal circulating 25(OH)D precursors, routine TB medication may cause a functional vitamin D shortage at the cellular level.

## Conclusion

Beyond VDR activation, vitamin D signaling represents a critical host-directed mechanism in tuberculosis, integrating tightly regulated enzymatic metabolism within immune and epithelial cells. Genetic variation in key vitamin D–metabolizing enzymes—including CYP2R1, CYP27A1, CYP27B1, and CYP24A1—may substantially influence the intracellular availability of active vitamin D, antimicrobial peptide induction, cytokine balance, and macrophage effector function. These findings highlight that circulating 25-hydroxyvitamin D [25(OH)D] levels do not necessarily reflect functional vitamin D activity at sites of infection, providing a plausible explanation for the inconsistent outcomes observed in vitamin D supplementation trials, particularly in individuals with metabolic comorbidities such as diabetes mellitus.

Collectively, current evidence supports a shift from uniform supplementation strategies toward host-directed, precision-based approaches that integrate genetic profiling, metabolic regulation, and immunological biomarkers. Although direct evidence in tuberculosis–diabetes populations remains limited, the convergence of metabolic and immune pathways underscores a critical area for future investigation. Targeting vitamin D metabolic pathways therefore represents a promising strategy to enhance antimycobacterial immunity and improve clinical outcomes. Future studies should prioritize genetically stratified and mechanistically informed interventions to identify patient populations most likely to benefit from vitamin D–based therapies.

## Data Availability

Data sharing not applicable to this article as no datasets were generated or analysed during the current study.
